# Dynamic observation of reductive and oxidative hydroxylation of CoO_*x*_ nanostructures in water vapor

**DOI:** 10.1093/nsr/nwag085

**Published:** 2026-02-09

**Authors:** Xiaoyuan Sun, Dongqing Wang, Rankun Zhang, Xiaoyu Liang, Le Lin, Rongtan Li, Rentao Mu, Qiang Fu

**Affiliations:** School of Chemistry, Dalian University of Technology, Dalian 116024, China; State Key Laboratory of Catalysis, Dalian Institute of Chemical Physics, Chinese Academy of Sciences, Dalian 116023, China; University of Chinese Academy of Sciences, Beijing 100049, China; State Key Laboratory of Catalysis, Dalian Institute of Chemical Physics, Chinese Academy of Sciences, Dalian 116023, China; School of Chemistry, Dalian University of Technology, Dalian 116024, China; State Key Laboratory of Catalysis, Dalian Institute of Chemical Physics, Chinese Academy of Sciences, Dalian 116023, China; State Key Laboratory of Catalysis, Dalian Institute of Chemical Physics, Chinese Academy of Sciences, Dalian 116023, China; University of Chinese Academy of Sciences, Beijing 100049, China; Interdisciplinary Research Center for Biology and Chemistry, Liaoning Normal University, Dalian 116029, China; State Key Laboratory of Catalysis, Dalian Institute of Chemical Physics, Chinese Academy of Sciences, Dalian 116023, China; State Key Laboratory of Catalysis, Dalian Institute of Chemical Physics, Chinese Academy of Sciences, Dalian 116023, China; School of Chemistry, Dalian University of Technology, Dalian 116024, China; State Key Laboratory of Catalysis, Dalian Institute of Chemical Physics, Chinese Academy of Sciences, Dalian 116023, China

**Keywords:** oxide surface, high-pressure scanning tunneling microscopy, redox reaction, reaction front, hydroxylation

## Abstract

Water plays important roles in many energy chemistry and catalysis processes and yet atomic-scale understanding of water–solid interactions in water-involved interfacial processes still remains underexplored. Here, combining high-pressure scanning tunneling microscopy and theoretical calculation we have visualized and elucidated both oxidation of CoO bilayers and reduction of CoO_2_ trilayers in water atmospheres. CoO bilayers are readily hydroxylated to Co(OH)_2_ with slight Co oxidation at 10^−8^ mbar H_2_O. At the CoO_2−_*_x_* surface, containing both CoO and CoO_2_ domains, hydroxylation of CoO produces a metastable Co(OH)_2_-CoO_2−_*_x_* interface, where H_2_O assists oxygen desorption from interfacial CoO_2_ and further hydroxylation of newly formed CoO. The dynamic Co(OH)_2_-CoO_2−_*_x_* reaction front drives unusual reductive hydroxylation of CoO_2−_*_x_* into Co(OH)_2_ under mbar-level H_2_O. Both CoO_2_ reduction through H_2_O-assisted oxygen desorption and CoO oxidation via H_2_O dissociative adsorption reveal a dynamic redox mechanism for water–oxide interactions.

## Teaser

Atomic-scale observations dynamically capture water-driven reductive and oxidative hydroxylation of supported cobalt oxide nanolayers with different oxidation states, revealing a dynamic redox mechanism in water-oxide interactions.

## INTRODUCTION

Water (H_2_O) is a ubiquitous molecule in a variety of reaction systems catalyzed by metal oxides, such as the water-gas shift reaction [1,2], oxygen evolution reaction [3–5], and syngas conversion reactions [6,7]. H_2_O can adsorb on metal oxides in the form of H_2_O*-M or M-OH*+MO-H*, i.e. molecular or dissociative adsorption [8–11]. Both adsorption mechanisms have been observed on many oxide surfaces, such as ZnO(10${\bar{\vphantom{1}} {1}0}$) [8,12], CeO_2_ [13], Fe_3_O_4_ [14], and MgO(001)/Ag(001) [15]. It is recognized that surface defect sites including oxygen vacancies and coordinatively unsaturated metal sites (CUSs) are beneficial to dissociative adsorption of H_2_O, leading to hydroxylation of many oxide surfaces [16–18]. Furthermore, H_2_O can adsorb on surfaces of TiO_2_(110), RuO_2_(110), and MgO(100) in both molecular and dissociative forms. The preferred adsorption state is governed by factors including surface structure, defects, and H_2_O coverage [19–21].

Surface adsorption of H_2_O also induces significant structural changes of working catalysts. It has been demonstrated that the interaction between H_2_O and oxides can facilitate the formation of surface hydroxyl groups and in some cases may even drive the conversion of oxide surfaces into catalytically active hydroxide structures [5,22]. For example, in electrocatalytic reaction environments H_2_O has been reported to trigger structural transformation of CoO, leading to the formation of CoO_2−_*_x_*(OH)*_x_* active phase [5]. In NO*_x_* storage and reduction reactions, the presence of H_2_O can enhance the performance of Pt/Ba/Al_2_O_3_ catalysts by supplying and stabilizing surface OH species [23]. In addition, the interaction between H_2_O and metal oxides often affects dispersion and stability of supported metals atop [1,24–26], co-adsorption of other molecules [27], and performance of oxide-catalyzed reactions. For instance, H_2_O can promote the dispersion of Ag particles on *γ*-Al_2_O_3_ [25] and Rh on *α*‑Fe_2_O_3_(1${\bar{\vphantom{1}} {1}}$02) [28]. H_2_O in the form of dissociative adsorption enhances co-adsorption of CO on CoO/Pt(111) and alters the distribution of adsorbed CO on rutile TiO_2_(110) [27,29]. Although a number of studies have elucidated the adsorption states of water and its catalytic role on the oxide surfaces, the direct observation of water-induced dynamic restructuring of oxides is still lacking, especially in a water vapor atmosphere and at the atomic scale.

Co-based oxides (CoO*_x_*) exhibit outstanding catalytic performance in a variety of reactions, e.g. CO_2_ hydrogenation reaction, water-gas shift reaction, oxygen evolution reaction, and hydrogen evolution reaction [30–32], owing to their multiple oxidation states. Notably, interaction with H_2_O molecules induces complex dynamic changes of the surface structure and electronic states of CoO*_x_*, such as the formation of hydroxylated structures and changes in valence states, all of which are closely linked to their catalytic activity [33,34]. In recent years, researchers have advanced the understanding of interfacial interactions between H_2_O and oxide surfaces by constructing model catalysts such as ultrathin CoO*_x_* films and Co oxide nanoislands [27,35,36]. The structural dynamics of well-defined Co oxide nanoislands on a metal surface have been investigated by surface science methods and theoretical calculations [37]. Nevertheless, the detailed mechanisms underlying H_2_O-mediated structural evolution and the key factors controlling the redox processes of Co-based oxides remain incompletely understood.

In this work, comparative studies of the structural evolution of CoO and CoO_2−_*_x_* (0 < *x* < 1) overlayers were performed by high-pressure scanning tunneling microscopy (HP-STM), X-ray photoelectron spectroscopy (XPS), and density functional theory (DFT) calculation. For CoO films containing only Co–O bilayer structures, exposure to H_2_O leads to a transformation into HO–Co–OH trilayers (Co(OH)_2_), accompanied by oxidation of Co ions. Notably, this hydroxylation process is not restricted to low-coordination Co edge sites; instead, our results show that H_2_O can be activated on the CoO surface. For CoO_2−_*_x_* film containing Co–O bilayer and O–Co–O trilayer (CoO_2_) domains, CoO first transforms into Co(OH)_2_ to form a Co(OH)_2_-CoO_2−_*_x_* interface. The reaction front formed between the unreacted CoO_2−_*_x_* domain and the newly generated Co(OH)_2_ domain then drives the further reduction of CoO_2_ into CoO, eventually transforming the entire CoO_2−_*_x_* film into Co(OH)_2_. We demonstrate clearly that supported oxide nanostructures can be either reductively or oxidatively hydroxylated by water, depending critically on their initial structural state. When H_2_O adsorption increases the coordination number of Co in CoO*_x_*, it acts as an oxidant. In contrast, when H_2_O adsorption promotes the removal of lattice oxygen from CoO*_x_*, it functions as a reductant. By establishing a direct link between oxide structure, reaction front dynamics, and the dual redox role of water, our study provides a new mechanistic framework for understanding water-driven redox reactions in oxide surfaces.

## RESULTS AND DISCUSSION

### Construction of CoO and CoO_2−_*_x_* nanostructures on Pt(111)

Cobalt oxide (CoO*_x_*) overlayers on Pt(111) were prepared by depositing metallic Co in 5 × 10^−8^ mbar O_2_ at room temperature (RT) and subsequently annealing at 573 K in the same atmosphere [38]. The STM image in Fig. S1a shows that the moiré pattern and surface atomic structure of 1 monolayer (ML) CoO film grown on Pt(111) are in good agreement with those of monolayer CoO(111) on Pt(111), containing a top O layer and a bottom Co layer (Co–O bilayer) [39]. After exposing the 1 ML CoO/Pt(111) surface to 5 × 10^−6^ mbar O_2_ at RT for 10 min, the sample surface shows the appearance of bright domains with a √3 × √3 *R*30° superstructure (inset in Fig. S1b), which agrees with the O–Co–O trilayer structure reported by Fester *et al.* [40]. A dark domain marked by the white dashed line in Fig. S1b shows the same moiré pattern as CoO. Meanwhile, the gaps (marked by a blue dashed circle in Fig. S1b) between CoO_2_ domains are also attributed to the unoxidized CoO regions. As a result, treating the 1 ML CoO/Pt(111) surface in 10^−6^ mbar O_2_ at RT can form an oxidized surface composed of both CoO and CoO_2_ domains, marked as CoO_2−_*_x_*/Pt(111) (0 < *x* < 1). It should be noted that for different scanning areas on 1 ML CoO_2−_*_x_*/Pt(111), the proportions of CoO domains are different (Fig. S2). This means that the oxidation of CoO is non-uniform at the nanoscale.

XPS results (Fig. S1c and d) show that the Co 2*p*_3/2_ peak shifts from 778.9 to 779.3 eV after the oxidation of 1 ML CoO/Pt(111) in 5 × 10^−6^ mbar O_2_ at RT for 10 min. In addition, the O 1*s* peak area increases and the O/Co atomic ratio also increases from 1.0 to 1.9. Following the oxidation of CoO into CoO_2−_*_x_*, the O 1*s* XPS peak shifts to a higher binding energy (BE) position (Fig. S1d). Meanwhile, a larger full width at half-maximum is observed in the spectrum, indicating the presence of multiple components. It has been found that the surface oxygen (upper layer oxygen) exhibits a lower BE compared with the interface oxygen (lower layer oxygen) in the O–Co–O trilayer structure [40]. The deconvoluted components (Fig. S3) show that the BEs of the surface and interface oxygen are 529.3 and 530.2 eV, respectively, and the peak at 531.7 eV is attributed to hydroxylated surface oxygen. Thus, 1 ML CoO/Pt(111) after oxidation can be marked as 1 ML CoO_1.9_/Pt(111). These XPS results are in line with STM observations confirming the formation of a large portion of CoO_2_ domains after O_2_ treatment.

### Hydroxylation-induced oxidation of CoO in H_2_O

**Figure 1.**
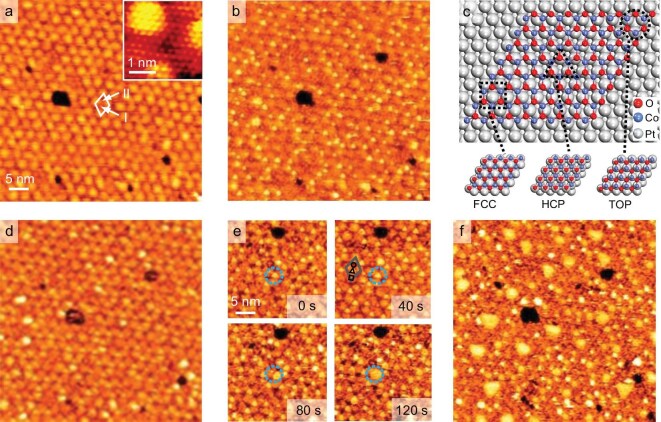
*In-situ* STM images of the hydroxylation of 1 ML CoO/Pt(111) under H_2_O atmospheres of 8 × 10^−8^ to 1 × 10^−5^ mbar. (a and b) *In-situ* STM images of an as-prepared 1 ML CoO/Pt(111) surface (a) and the surface in 8 × 10^−8^ mbar H_2_O (b). Inset in (a) shows the atomic-resolution image of the CoO surface. The moiré unit cell in (a) is marked by the white parallelogram and two kinds of hollow sites in the cell are marked by I and II. (c) The ball model of the moiré unit cell of CoO/Pt(111). The FCC, HCP, and TOP domains in (c) are marked by a square, triangle, and circle, respectively. (d–f) *In-situ* STM images of an as-prepared 1 ML CoO/Pt(111) surface in 5 × 10^−7^ (d), 2 × 10^−6^ (e), and 1 × 10^−5^ (f) mbar H_2_O. The growth of a bright dot in (e) is marked by blue dashed circles. The moiré unit cell in (e) is marked by a gray parallelogram. The FCC, HCP, and TOP domains in (e) are marked by a square, triangle, and circle, respectively. STM parameters: *V_t_* = 1.25 V, *I_t_* = 0.1 nA.

*In-situ* HP-STM was employed to investigate the structural evolution of CoO under H_2_O atmosphere at RT. Figure 1a and b shows that bright dots appear on the 1 ML CoO/Pt(111) surface under 10^−8^ mbar H_2_O but only at one type of hollow site (marked by I in Fig. 1a) of the moiré unit cell (marked by the white parallelogram in Fig. 1a). It is proposed that the moiré unit cell of CoO/Pt(111), as shown in Fig. 1c, contains three high-symmetry domains in which the Co atoms adopt on-top (TOP), hexagonal close-packed (HCP), or face-centered cubic (FCC) stacking relative to the interfacial Pt atoms [39]. The identification of the domains in STM images can be achieved through the intentional creation of O vacancies on the CoO surface (Fig. S4) [39,41]. Accordingly, the location of these bright dots can be attributed to the HCP domain, meaning that the HCP domain is more active for H_2_O adsorption than other sites. As the H_2_O pressure increased to 10^−6^–10^−5^ mbar, some bright dots grew into bright patches (Fig. 1d–f, for the full series see Movie S1).

**Figure 2.**
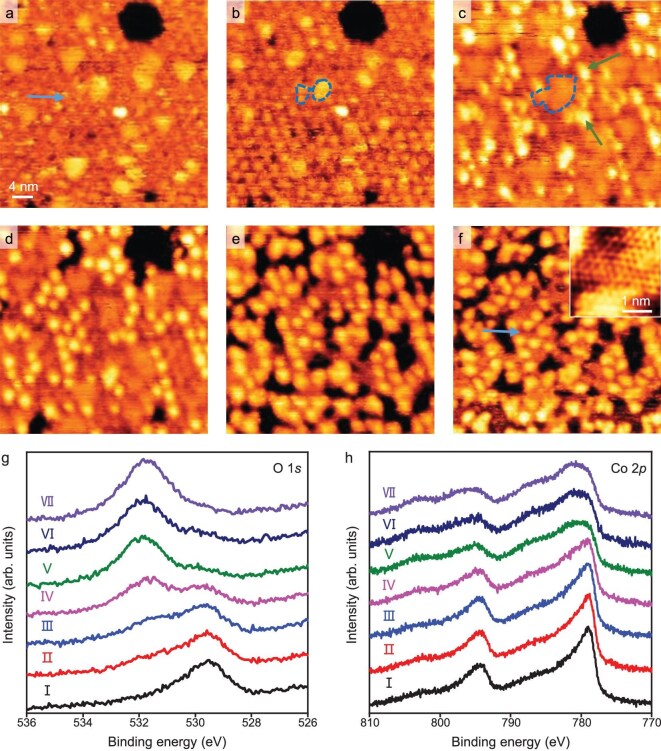
*In-situ* STM images of the hydroxylation of 1 ML CoO/Pt(111) under H_2_O atmospheres of 1 × 10^−5^ to 2 mbar. (a) STM image of 1 ML CoO/Pt(111) exposed to 1 × 10^−5^ mbar H_2_O and then pumped to ultra-high vacuum for imaging. (b–f) *In-situ* STM images of 1 ML CoO/Pt(111) in 1 × 10^−4^ (b), 5 × 10^−4^ (c), 1 × 10^−3^ (d), 0.3 (e), and 2 (f) mbar H_2_O. The inset in (f) shows the atomic-resolution image of the Co(OH)_2_ surface. The growth of two bright patches in (b) and (c) are marked by blue dashed lines. (g and h) O 1*s* (g) and Co 2*p* (h) XPS spectra of 1 ML CoO/Pt(111) surface at seven stages of H_2_O dissociative adsorption. Ⅰ: pristine, Ⅱ: 5 × 10^−7^, Ⅲ: 5 × 10^−6^, Ⅳ: 5 × 10^−5^, Ⅴ: 5 × 10^−4^, Ⅵ: 5 × 10^−3^, and Ⅶ: 6 mbar H_2_O for 10 min at RT. STM parameters: *V_t_* = 1.25 V, *I_t_* = 0.1 nA.

The bright features induced by H_2_O adsorption on 1 ML CoO/Pt(111) remain unchanged after pumping the H_2_O pressure from 1 × 10^−5^ mbar to ultra-high vacuum (Fig. 2a). These bright patches continue to expand (marked by blue dashed lines in Fig. 2b and c) under 10^−4^ mbar H_2_O. Further, some brighter features (marked by green arrows in Fig. 2c) start to appear at the edges of the bright patches. Under 2 mbar H_2_O, the mentioned brighter features become dominant on the surface (Fig. 2e and f, for the full series see Movie S2). Meanwhile, the depth profile of the same surface regions (Fig. S5) in Fig. 2a and f shows that the original intact oxide film becomes fragmented after exposure to an mbar-level H_2_O atmosphere (marked by blue arrows in Fig. 2a and f).

The structural evolution of 1 ML CoO/Pt(111) after exposure to H_2_O was also investigated by XPS. Figure 2g shows that a hydroxyl O 1*s* signal appears and its amount increases while that of lattice O decreases gradually when the partial pressure of H_2_O increases. This means that H_2_O adsorbs on the CoO/Pt(111) surface in dissociative form. Meanwhile, the binding energy of the Co 2*p*_3/2_ peak shifts from 778.9 to 780.7 eV and the satellite peak of Co 2*p*_3/2_ becomes stronger (Fig. 2h), which is consistent with the transformation of CoO to Co(OH)_2_ [42]. After treatment in 6 mbar H_2_O, the lattice O signal almost disappears completely and the O/Co atomic ratio increases to 2.0 (Fig. 2g and h), indicating the nearly complete transformation of CoO to Co(OH)_2_. This is consistent with the brighter features observed earlier in Fig. 2a–f, which correspond to the emerging Co(OH)_2_ domains. It should be noted that, based on XPS analysis, the presence of trace amounts of CoOOH cannot be completely excluded. However, its contribution is minor compared to the dominant Co(OH)_2_. Moreover, the Co 2*p* peak positions of the ultrathin Co(OH)_2_ film grown on Pt(111) differ from those reported for Co(OH)_2_ foil [42,43]. Bader charge analysis [44] was performed to evaluate the oxidation state of Co atoms in both CoO and Co(OH)_2_ structures (Fig. S6 and Table S1). In the CoO/Pt(111) system, the Bader charge of Co is 1.16*e* (corresponding to a nominal oxidation state of 1.77), while in the Co(OH)_2_/Pt(111) system it increases to 1.33*e* (nominal oxidation state of 2.05). Thus, Co undergoes further oxidation during the transformation from CoO to Co(OH)_2_. The oxidation is mainly attributed to the increase in the coordination number of the Co atom in Co(OH)_2_, which reaches 6-fold coordination. Compared with a single O^2−^ ligand, two OH^−^ ligands possess stronger electron-withdrawing capability, thereby leading to a more pronounced reduction in the electron density of the Co center.

**Figure 3.**
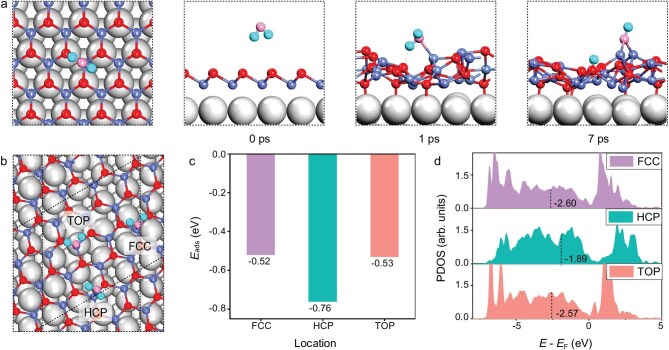
Theoretical analysis of the adsorption and dissociation of H_2_O on CoO/Pt(111). (a) AIMD simulations illustrating the dynamic behavior and structural evolution of H_2_O adsorption on the CoO/Pt(111) surface. (b) Structural models of H_2_O adsorption sites in the three domains of CoO/Pt(111). (c) Adsorption energies of H_2_O on the three domains of CoO/Pt(111). (d) Projected density of states (PDOS) for d-orbitals of Co in the three domains of CoO/Pt(111). Inserted values denote the positions of the d-band center. Pt: grey; Co: light blue; O: red and pink; and H: cyan.

To gain deeper insight into the dynamic evolution of the CoO/Pt(111) interface under a H_2_O atmosphere, *ab initio* molecular dynamics (AIMD) simulations and DFT calculations were performed. The AIMD results (Fig. 3a) reveal that as H_2_O molecules approach the CoO/Pt(111) surface, the CoO structure becomes more disordered. H_2_O molecules can coordinate with these exposed Co sites and simultaneously form hydrogen bonds with adjacent lattice oxygen atoms, thereby synergistically promoting H_2_O dissociation and formation of surface hydroxyl species. We adopted a previously reported CoO-(√13 × √13)/Pt(111)-(4 × 4) model (Fig. 3b) and examined the adsorption behavior of H_2_O molecules on FCC, HCP, and TOP domains [45]. DFT calculations (Fig. 3c) show that the HCP domain exhibits the strongest H_2_O adsorption (−0.76 eV), compared to −0.52 eV for FCC and −0.53 eV for TOP, indicating a thermodynamic preference. Moreover, the Co atoms in this region have the highest d band center (*ε*_d_ = −1.89 eV, Fig. 3d) [46], suggesting the strongest interaction with H_2_O molecules. As a result, we conclude that the HCP domain of the moiré unit cell of CoO/Pt(111) is the active site for the dissociative adsorption of H_2_O and formation of Co(OH)_2_ domains, which is consistent with experimental observations showing higher H_2_O adsorption activity on HCP domains compared to other sites.

### Hydroxylation-induced reduction of CoO_1.9_ in H_2_O

**Figure 4.**
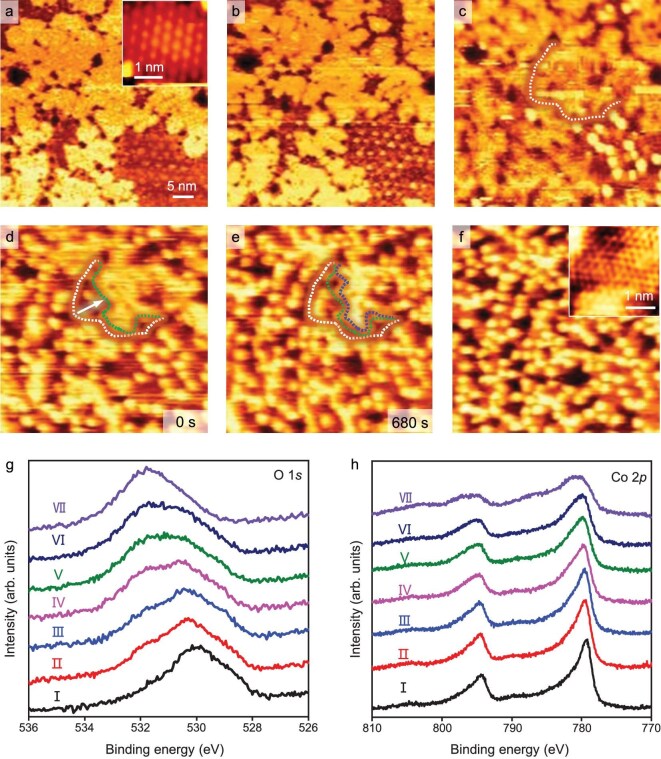
*In-situ* STM images of the hydroxylation of 1 ML CoO_1.9_/Pt(111) under H_2_O atmospheres of 1 × 10^−4^ to 2 mbar. (a–f) *In-situ* STM images of 1 ML CoO_1.9_/Pt(111) (a) in 1 × 10^−4^ (b), 0.02 (c), 0.4 (d and e), and 2 (f) mbar H_2_O. The insets in (a) and (f) show the atomic-resolution images of the CoO_2_ and Co(OH)_2_ surfaces. The transformation fronts in (c–e) are marked by white, green, and blue dashed lines and the transformation directions of the fronts are marked by the white arrow. (g and h) O 1*s* (g) and Co 2*p* (h) XPS spectra of 1 ML CoO_1.9_/Pt(111) at seven stages of H_2_O dissociative adsorption. Ⅰ: pristine, Ⅱ: 5 × 10^−7^, Ⅲ: 5 × 10^−6^, Ⅳ: 5 × 10^−5^, Ⅴ: 5 × 10^−4^, Ⅵ: 5 × 10^−3^, and Ⅶ: 6 mbar H_2_O for 10 min at RT. STM parameters: *V_t_* = 1.25 V, *I_t_* = 0.1 nA.

The structural evolution of 1 ML CoO_1.9_/Pt(111) under H_2_O atmospheres is illustrated in Fig. 4. Under 10^−4^–10^−2^ mbar H_2_O atmosphere, the brighter features begin to appear on CoO domains (Fig. 4a–c). This indicates that the CoO domains are transformed into Co(OH)_2_ and thus the initial CoO-CoO_2_ interfaces shown in Fig. 4a are transformed into Co(OH)_2_-CoO_2_ interfaces (Fig. 4c). Under 0.1 mbar H_2_O atmosphere, the newly formed CoO_2_-Co(OH)_2_ interfaces can act as reaction fronts for further structural transformation (marked by the white dashed line in Fig. 4c–e, for the full series see Movie S3), and move to the interior of CoO_2_ domains until the whole surface is covered by the brighter features (Fig. 4f). The scanning area on 1 ML CoO_1.9_/Pt(111) with lower CoO proportion (<10%) was also investigated in the same H_2_O atmosphere, and a similar transformation process was observed (Fig. S7). XPS results of the evolution processes of 1 ML CoO_1.9_/Pt(111) after exposure to H_2_O at elevated pressures are shown in Fig. 4g and h. After H_2_O exposure, a hydroxyl O 1*s* signal appears, accompanied by a gradual decrease in the lattice O signal. At the same time, the Co 2*p* peak shifts toward higher binding energy. The distinctive feature is that the O 1*s* peak intensity of CoO_1.9_ film does not show an obvious increase after H_2_O exposure, and the O/Co atomic ratio remains consistently around 2.0.

**Figure 5.**
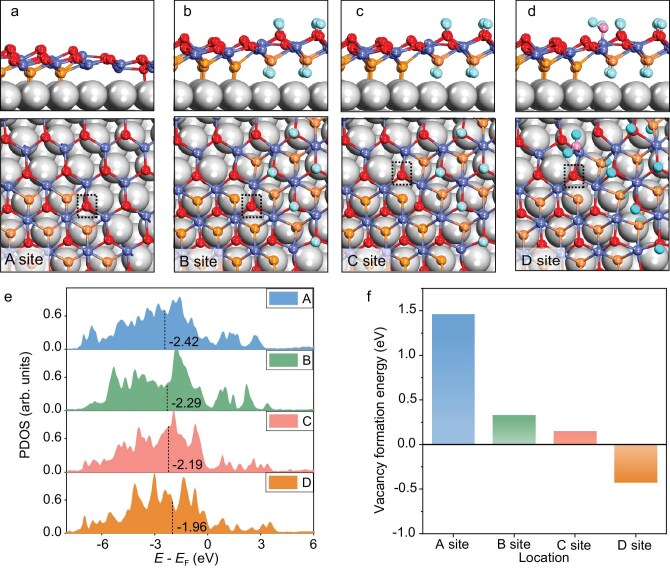
Nature of the structural reduction in CoO_2_/Pt(111). (a–d) Configurations (top and side views) of CoO-CoO_2_ (a), CoO_2−_*_x_*-Co(OH)_2_ (b and c), and CoO_2−_*_x_*-Co(OH)_2_ with H_2_O adsorbed (d) on the oxide surface, all supported on Pt(111). (e) PDOS of the interfacial O atoms are highlighted in (a–d) with the p band centers given. (f) Oxygen vacancy formation energies at the four interfacial O sites indicated in (a–d). Pt: grey; Co: light blue; O: red, orange, and pink; and H: cyan.

To elucidate the structural evolution of CoO_2_/Pt under H_2_O atmosphere, we performed DFT calculations on CoO_2_-CoO and CoO_2−_*_x_*-Co(OH)_2_ interfacial models supported on Pt(111) (Fig. 5a–d). Oxygen vacancy formation energies were evaluated at the interfacial sites of the CoO_2_-CoO and CoO_2−_*_x_*-Co(OH)_2_ structures. For the CoO_2_-CoO model, we constructed an oxygen vacancy by removing an O atom (A site) at the CoO_2_-CoO interface. For the CoO_2−_*_x_*-Co(OH)_2_ model, oxygen vacancy was introduced at the CoO_2_-Co(OH)_2_ interface (B site) and the interface involving CoO, Co(OH)_2_, and CoO_2_ (C site). Electronic structure analysis shows that the O 2*p* band centers at sites A, B, and C are −2.42, −2.29, and −2.19 eV [47], respectively (Fig. 5e), reflecting increased interfacial oxygen activity as the interface transforms from CoO_2_-CoO to CoO_2−_*_x_*-Co(OH)_2_. However, the oxygen vacancy formation energies on the CoO/Pt(111) surface and the three interface sites A, B, and C are 1.81, 1.46, 0.33, and 0.15 eV, respectively (Fig. 5f). All positive values indicate that the formation of these vacancies is still thermodynamically unfavorable.

In contrast, Fig. 5d illustrates significant lattice distortion at the CoO_2−_*_x_*-Co(OH)_2_ interface, facilitating preferential adsorption of H_2_O at the interfacial Co sites. Upon H_2_O adsorption, the oxygen vacancy formation energy at the neighboring oxygen site (D site) is reduced to −0.42 eV, indicating favorable vacancy formation. This is consistent with the upward shift of the O 2*p* band center at this site to −1.96 eV (Fig. 5f), which denotes enhanced oxygen activity. In addition, the Bader charge of Co ions from the Co(OH)_2_/Pt(111) surface is 1.33*e* (corresponding to a nominal oxidation state of 2.05), which is lower than that of 1.39*e* (nominal oxidation state of 2.30) in CoO_2_/Pt(111) (Fig. S6 and Table S1) [45]. The magnetic moment further supports this trend: CoO_2_/Pt(111) exhibits a moment of 0.00 μB, consistent with Co^3+^ electronic character, whereas Co(OH)_2_/Pt(111) shows a moment of 2.72 μB, characteristic of Co^2+^ (Table S1). These results indicate that the transformation from CoO_2_ to Co(OH)_2_ involves reduction of Co ions. Therefore, under H_2_O atmosphere the structural evolution of CoO_2−_*_x_*/Pt(111) proceeds via an initial transformation of the CoO domain into Co(OH)_2_, resulting in the formation of a CoO_2−_*_x_*-Co(OH)_2_ interface. At this interface, lattice oxygen atoms are activated by lattice distortion and interaction with H_2_O, promoting the reduction of CoO_2_ to CoO, which subsequently converts to Co(OH)_2_.

### Comparative hydroxylation processes of CoO and CoO_1.9_ overlayers

**Figure 6.**
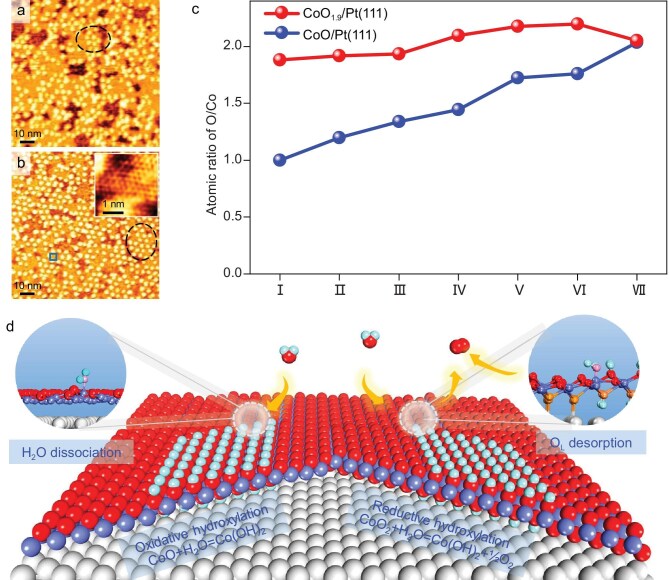
Comparisons of CoO/Pt(111) and CoO_2−_*_x_*/Pt(111) surfaces after exposure to H_2_O. (a and b) STM images of CoO_2−_*_x_*/Pt(111) (a) and CoO/Pt(111) (b) surfaces after exposure to 7 mbar H_2_O. Inset in (b) is the atomic structure of the area marked by the blue box. The brighter features in (a) and (b) are marked by a black oval. STM parameters in (a and b): *V_t_* = 1.25 V, *I_t_* = 0.1 nA; inset in (b): *V_t_* = 0.2 V, *I_t_* = 0.3 nA. (c) O/Co atomic ratios of 1 ML CoO/Pt(111) (blue) and CoO_1.9_/Pt(111) (red) at seven stages of H_2_O dissociative adsorption. Ⅰ: pristine, Ⅱ: 5 × 10^−7^, Ⅲ: 5 × 10^−6^, Ⅳ: 5 × 10^−5^, Ⅴ: 5 × 10^−4^, Ⅵ: 5 × 10^−3^, and Ⅶ: 6 mbar H_2_O for 10 min at RT. (d) Schematic of reductive and oxidative hydroxylation of CoO*_x_* nanostructures in H_2_O atmosphere. Pt: grey; Co: light blue; O: red, orange and pink; H: cyan.

Comparisons of the CoO/Pt(111) and CoO_1.9_/Pt(111) surface structures after exposure to 7 mbar H_2_O atmosphere are shown in Fig. 6. Both hydroxylated 1 ML CoO/Pt(111) and CoO_1.9_/Pt(111) surfaces have the same bright features with a similar hexagonal arrangement (marked by black ovals in Fig. 6a and b) after exposure to 7 mbar H_2_O. Line profiles from STM images show that the holes on the two hydroxylated samples have the same average depth of 1.53 ± 0.4 Å (Fig. S8b and f) and bright features with a similar height of 1.28 ± 0.1 Å (Fig. S8d and h). These indicate that the CoO/Pt(111) and CoO_1.9_/Pt(111) surfaces are transformed into the same Co(OH)_2_/Pt(111) surface structure after exposure to 7 mbar H_2_O atmosphere. The atomic structure of Co(OH)_2_/Pt(111) is shown in the inset of Fig. 6b, having a similar structure to that of CoO/Pt(111). The interatomic distance of Co(OH)_2_/Pt(111) is determined to be 2.94 ± 0.2 Å (Fig. S9), which is smaller than that of CoO/Pt(111) (3.1 ± 0.1 Å). This is consistent with the observed decrease in the coverage of the overlayer on Pt(111) from 97% (Fig. 1a) to 91% (Fig. 2f) after transforming into Co(OH)_2_.

The variation of the O/Co atomic ratio for CoO and CoO_1.9_ overlayers on Pt(111) after exposure to H_2_O vapor is shown in Fig. 6c. In the transformation process of CoO to Co(OH)_2_, the O/Co atomic ratio increases from 1.0 to 2.0 due to the dissociative adsorption of H_2_O. The hydroxylation reaction equation is as follows: CoO + H_2_O → Co(OH)_2_. In contrast, for the transformation of CoO_1.9_ to Co(OH)_2_, the O/Co atomic ratio is always estimated to be ∼2.0. Considering that both CoO_2_ and Co(OH)_2_ films have the same O/Co ratio of 2.0, the reaction between CoO_2_ and H_2_O to form Co(OH)_2_ may involve the release of O_2_ from CoO_2_, which should derive from the decomposition of CoO_2_ at the CoO_2−_*_x_*-Co(OH)_2_ interface induced by H_2_O adsorption. Accordingly, the hydroxylation reaction equation is described as follows: 2CoO_2_ + 2H_2_O → 2Co(OH)_2_ + O_2_.

## CONCLUSION

We find that both CoO and CoO_2−_*_x_* nanostructures formed on Pt(111) undergo transformation into the same Co(OH)_2_ structure under mbar-level H_2_O vapor atmospheres. For the CoO/Pt(111) surface, the HCP domain of the moiré unit cell exhibits higher activity in transformation into Co(OH)_2_, accompanied by Co ion oxidation via a hydroxylation reaction. The corresponding hydroxylation reaction equation of CoO with H_2_O is as follows: CoO + H_2_O → Co(OH)_2_. For the CoO_1.9_/Pt(111) surface containing CoO and CoO_2_ domains, the transformation to Co(OH)_2_ occurs in two steps. Initially, CoO domains transform into Co(OH)_2_, forming a new Co(OH)_2_-CoO_2−_*_x_* interface. Assisted by H_2_O adsorption, CoO_2_ readily releases oxygen and is reduced to CoO under water atmosphere. The resulting CoO further converts into Co(OH)_2_, and the Co(OH)_2_-CoO_2−_*_x_* interface acts as the reaction front that drives the progressive transformation across the surface. The O_2_ release and Co ion reduction during the process can be represented by the reaction equation: 2CoO_2_ + 2H_2_O → 2Co(OH)_2_ + O_2_. This work provides atomistic insights into the H_2_O-induced reconstruction pathways of CoO*_x_*, and offers mechanistic guidance for the rational design of oxide-based catalysts in water-containing environments.

## METHODS

All experiments in this work were performed in an Omicron multiprobe ultra-high vacuum (UHV) system, which consists of a preparation chamber (base pressure <4 × 10^−10^ mbar), a spectroscopic analysis chamber (base pressure <1 × 10^−10^ mbar), and a microscopic chamber (base pressure <3 × 10^−10^ mbar). The spectroscopic chamber is equipped with an X-ray photoelectron spectroscope with a hemispherical analyzer (Omicron EA125 5-channeltron). The microscopic chamber is equipped with a high-pressure scanning tunneling microscope (SPECS). All XPS experiments were conducted with Al K*α* source (1486.6 eV). STM images were obtained via a tungsten tip at RT. Peak fitting of the measured XPS spectra was performed using Shirley background and Gaussian–Lorentzian line shapes. Binding energies were calibrated based on Pt 4*f*_7/2_ (71.9 eV) peaks. Statistical analysis of the XPS fitting results in this work is presented in Table S2.

A clean Pt(111) surface was prepared by repeated cycles of Ar^+^ sputtering (1.7 kV, 8 × 10^−6^ mbar, 10 min) at RT, annealing at 800 K in 5 × 10^−7^ mbar O_2_ and at 1000 K in UHV for 10 min, respectively, until no impurities were detected by XPS. Deposition of Co was performed by evaporating Co slugs (Alfa Aesar, 99.995%) at 1483 K in a high-temperature evaporator (CreaTec). The pristine cobalt oxide film (CoO) with single-layer thickness on Pt(111) was obtained by reactive deposition of Co in 5 × 10^−8^ mbar O_2_ and subsequently annealing at 573 K in the same environment for 15 min. The partially oxidized cobalt oxide was prepared by exposing the CoO/Pt(111) mentioned above to 5 × 10^−6^ mbar O_2_ at RT for 10 min. Ar (Arkonic gases, 99.9999%) and O_2_ (Arkonic gases, 99.9999%) were dosed into the chambers via sapphire leak valves. O_2_ was purified using liquid nitrogen for 1 h before being introduced into the chambers. H_2_O was purified by repeated freeze-pump-thaw cycles for three times.

Spin-polarized DFT calculations were implemented using a plane-wave basis set in the Vienna *ab initio* simulation package (VASP 5.4) [48]. The exchange–correlation energy was treated using the Perdew–Burke–Ernzerhof (PBE) functional within the generalized gradient approximation [49]. The projector-augmented-wave pseudopotentials were utilized to describe the core electrons, and a cutoff energy of 400 eV was used for the plane-wave expansion. The following valence electron configurations were included in the self-consistent field calculations: Pt (5d^9^ and 6s^1^), Co (3d^8^ and 4s^1^), O (2s^2^ and 2p^4^), and H (1s^1^). In addition, the van der Waals (vdW) dispersion forces were corrected by the vdW-DF (optPBE) functionals, which showed a highly accurate description of oxides [50]. An on-site Hubbard term *U*_eff_ = *U* − *J* was added to address the open-shell d-electrons with 3.2 eV for Co [45]. The water-based reference state for the calculations has been adopted to avoid an incorrect description of the gas phase O_2_ with standard DFT methods [50]. The energies and residual forces were converged to 10^−5^ eV and 0.02 eV Å^−1^, respectively.

## Supplementary Material

nwag085_Supplemental_Files
