# Supplementary material for: Dynamic observation of reductive and oxidative hydroxylation of CoOx nanostructures in water vapor
Source: Natl Sci Rev. 2026 Feb 9;13(6):nwag085. doi: 10.1093/nsr/nwag085 (PMC13034533; doi:10.1093/nsr/nwag085)
Supplement: nwag085_Supplemental_Files [file nwag085_supplemental_files.zip › Supplementary data.pdf]

Supplementary Material

Dynamic Observation of Reductive and Oxidative Hydroxylation of CoO_x Nanostructures in Water Vapor

Xiaoyuan Sun[1,2,3,†], Dongqing Wang[2,†], Rankun Zhang[1,2], Xiaoyu Liang[2,3], Le Lin[4], Rongtan Li[2], Rentao Mu[2,*], Qiang Fu[1,2,*]

[1]School of Chemistry, Dalian University of Technology, Dalian, 116024, China

[2]State Key Laboratory of Catalysis, Dalian Institute of Chemical Physics, Chinese Academy of Sciences, Dalian, 116023, China

[3]University of Chinese Academy of Sciences, Beijing 100049, China

[4]Interdisciplinary Research Center for Biology and Chemistry, Liaoning Normal University, Dalian 116029, China

*Corresponding authors.

E-mail addresses: murt@dicp.ac.cn (R. Mu); qfu@dicp.ac.cn (Q. Fu).

METHODS

Model Constructions. We built three fundamental models to investigate the structural evolution of CoO_x/Pt(111) under H_2O atmosphere. The CoO/Pt(111) interface was modeled using a reported moiré superstructure, CoO-($\sqrt{13} \times \sqrt{13}$)/Pt(111)-($4 \times 4$), which amounts to placing a monolayer CoO film on a three-layer Pt(111) substrate, with the bottom Pt layers fixed. This relatively small supercell was chosen to keep the computational cost tractable while still preserving the essential structural characteristics of the experimentally observed CoO moiré pattern, including Co/Pt ratio, local coordination environment, and TOP, HCP, and FCC domains. We further verified that the electronic structure characteristics of the Co sites obtained from this model are consistent with those from the larger moiré supercell (Figure S10), confirming that the simplified model reproduces accurate relative activity trends among different Co sites. The CoO_2-CoO interface was constructed based on a CoO-($\sqrt{67} \times \sqrt{67}$)/Pt(111)-($\sqrt{84} \times \sqrt{84}$) moiré superstructure by inserting additional O atoms into the Co-Pt interlayer within the FCC domains of the surface, forming an oxidized CoO_2-CoO structure. The CoO_{2-x}-$Co(OH)_2$ interface was modeled by further introducing H and OH species into the surface O sites and the Co-Pt interlayer in the HCP domains of the CoO_2-CoO surface. For all structures, the CoO_x overlayer and the top two Pt layers were fully relaxed, while the bottom Pt layer were constrained. The ($3 \times 3 \times 1$), ($1 \times 1 \times 1$), and ($1 \times 1 \times 1$) k-point grids were employed for the CoO, CoO_2-CoO, and CoO_{2-x}-$Co(OH)_2$ models, respectively.

Ab initio molecular dynamics (AIMD). AIMD simulations were performed in the NVT (canonical) ensemble at 298 K using VASP with a Nosé–Hoover thermostat. The total simulation time was 7 ps with a time step of 1 fs.

Oxidation state calculations. The oxidation states of Co in the ultrathin films were estimated by calculating Bader charges and normalizing them to Bader charges of bulk compounds with known oxidation states [1]. For the CoO monolayer supported on Pt(111), the Bader charge of Co was 1.16 *e*. When normalizing to bulk $Co(OH)_2$ with Bader charge 1.31*e* and oxidation state of two, nominal oxidation states of 1.77 was obtained. For Co in supported $Co(OH)_2$ and CoO_2 films with Bader charges of 1.33 *e* and 1.39 *e*, a separate linear interpolation, which is accurate for Co with higher oxidation states, is employed. These values were normalized to the Bader charges of bulk $Co(OH)_2$,

1.31*e*, and CoOOH, 1.55*e* (bulk oxidation states of two and three), yielding film oxidation states of 2.05 and 2.30, respectively.

Formulae. (1) The energy level of each state was corrected to the Gibbs free energy (denoted here as the chemical potential, μ) by

$$\mu_{gas} = E_{elec} + ZPE + \delta H - TS + k_B T \ln \frac{P_{gas}}{P^o} \#(1)$$

where E_{elec} represents the electronic energy calculated by DFT at 0 K, *ZPE* is the zero-point energy, δH is the integral of the heat capacity, and *TS* corresponds to the entropy change. For gaseous species (μ_{gas}), the corrections were derived from the *NIST* database using the standard ideal-gas method, with consideration of the partial pressure of the gas (P_{gas}) [2,3]. The corrected values are listed in Table S3.

(2) The adsorption energy (E_{ads}) of H_2O is

$$E_{ads} = E_{H_2O*} - E_* - E_{H_2O} \#(2)$$

where E_{H_2O*}, E_* and E_{H_2O} are the energies of the H_2O adsorbed surface, clean surface and gaseous H_2O molecule.

(3) The formation energy of oxygen vacancy [$G_{vf}(O)$] was denoted by

$$G_{vf}(O) = E_{Vo} - E_{int} + \frac{1}{2}\mu_{O_2} \#(3)$$

where E_{Vo} and E_{int} are the total energies of the oxygen-defected and the intact surfaces. μ_{O_2} is the energy of gaseous O_2.

(4) The d band center of Co (ε_d) was defined as

$$\varepsilon_d = \frac{\int_{\varepsilon_{min}}^{\varepsilon_{max}} n_d(\varepsilon)\varepsilon \, d\varepsilon}{\int_{\varepsilon_{min}}^{\varepsilon_{max}} n_d(\varepsilon) \, d\varepsilon} \#(4)$$

(5) The p band center of O (ε_p) was defined as

$$\varepsilon_p = \frac{\int_{\varepsilon_{min}}^{\varepsilon_{max}} n_p(\varepsilon)\varepsilon \, d\varepsilon}{\int_{\varepsilon_{min}}^{\varepsilon_{max}} n_p(\varepsilon) \, d\varepsilon} \#(5)$$

The choice of descriptor corresponds to the nature of the active sites participating in each process.

Figures and tables

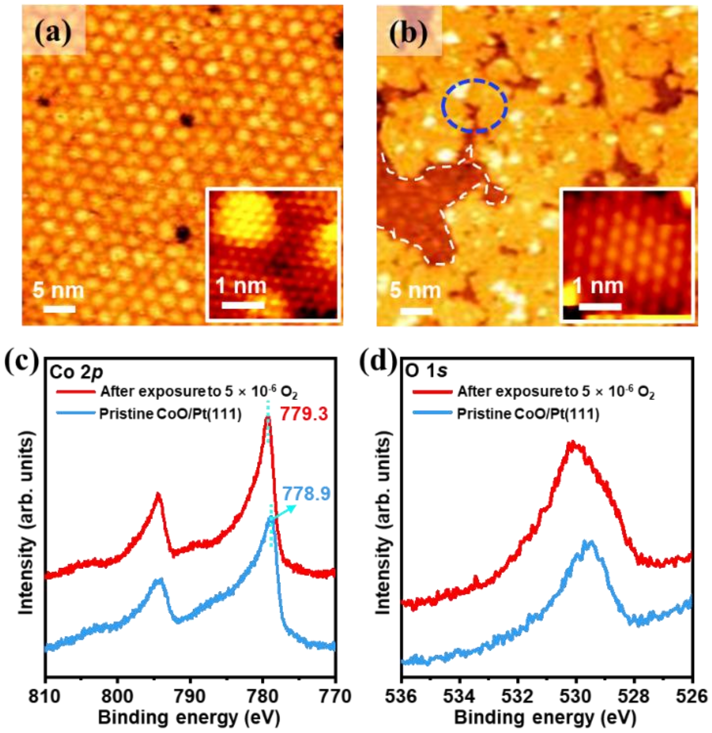

Figure S1. (**a–b**) STM images of 1 ML CoO on Pt(111) before (**a**) and after (**b**) the exposure to 5 $\times 10^{-6}$ mbar O_2 for 10 min at RT. Insets in (**a**) and (**b**) show the atomic resolution images of CoO and CoO_2 surfaces. The dark domain in (**b**) marked by white dash lines is from CoO domain. (**c–d**) Co 2*p* (**c**) and O 1*s* (**d**) XPS spectra of 1 ML CoO on Pt(111) before and after the exposure to 5 $\times 10^{-6}$ mbar O_2 at RT for 10 min. STM parameters: V_t = 2.0 V, I_t = 0.8 nA; Inset in (**a**): V_t = 0.1 V, I_t = 0.31 nA; Inset in (**b**): V_t = 0.88 V, I_t = 0.24 nA.

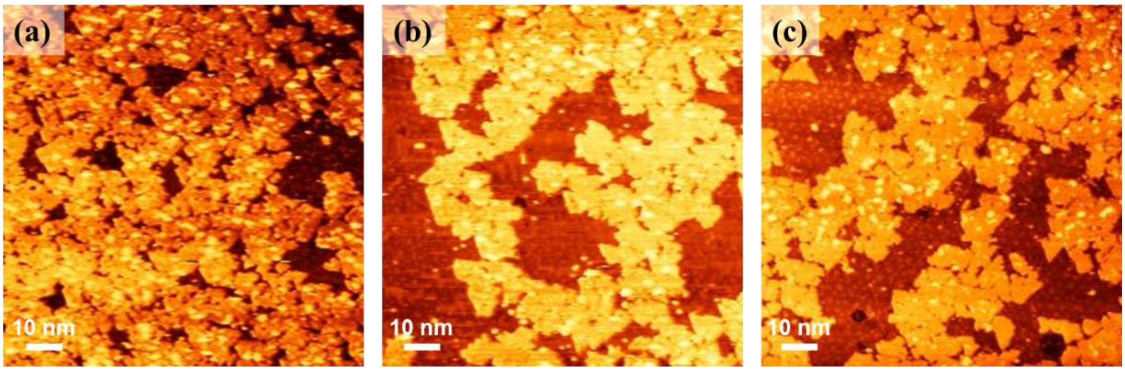

Figure S2. Three different scanning areas on same CoO_{2-x} film (O/Co = 1.9). STM parameter V_t = 1.25V, I_t = 0.1nA.

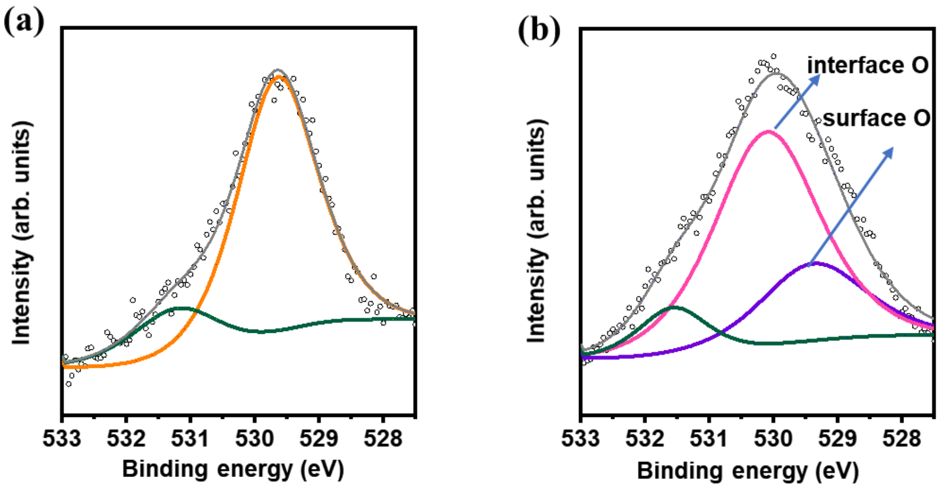

Figure S3. XPS O 1*s* spectra of the (a) CoO/Pt(111) and (b) CoO_{2-x}/Pt(111) surfaces. Fitting peaks of CoO in orange and green represent lattice oxygen and surface hydroxyl, respectively, and fitting peaks of CoO_{2-x} in purple, pink, and green represent surface lattice oxygen, interface lattice oxygen, and surface hydroxyl, respectively.

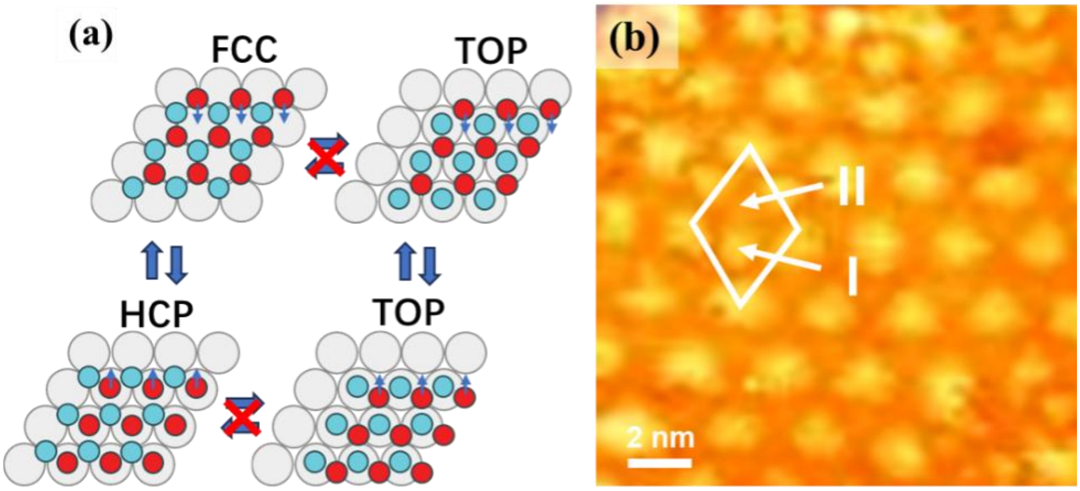

Figure S4. (a) Ball models of moiré structures. O: red; Co: blue; Pt: grey. (b) STM image of O dislocation triangle on CoO/Pt(111). STM parameter V_t = 1.25V, I_t = 0.1nA.

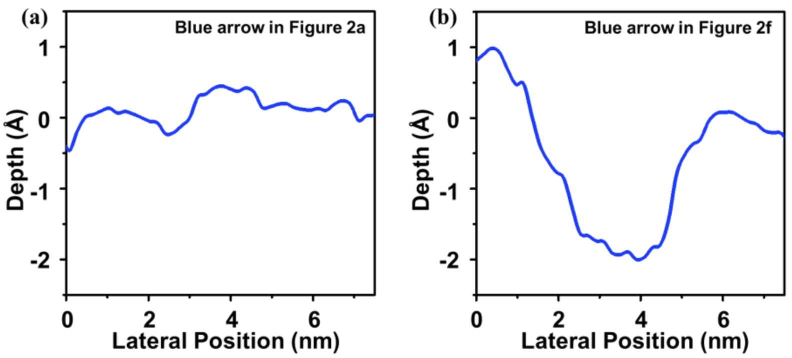

Figure S5. Line profile along the blue arrow in Figure 2a and 2f.

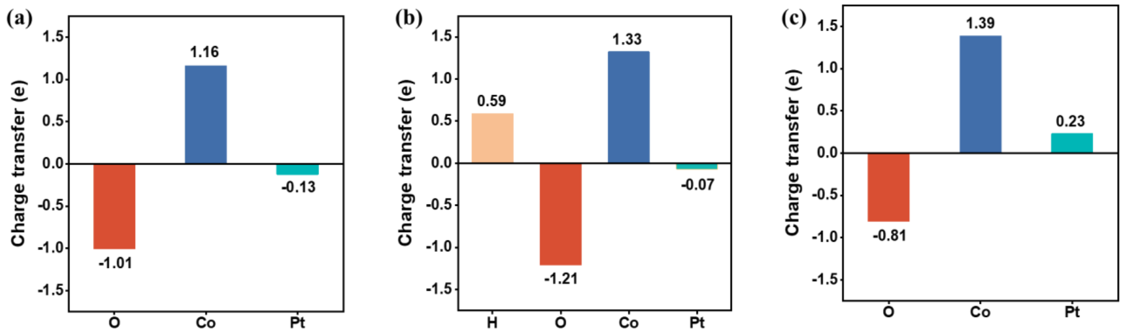

Figure S6. Bader charge transfer analysis for (a) CoO/Pt(111), (b)Co(OH)$_2$/Pt(111), and (c) CoO_2/Pt(111).

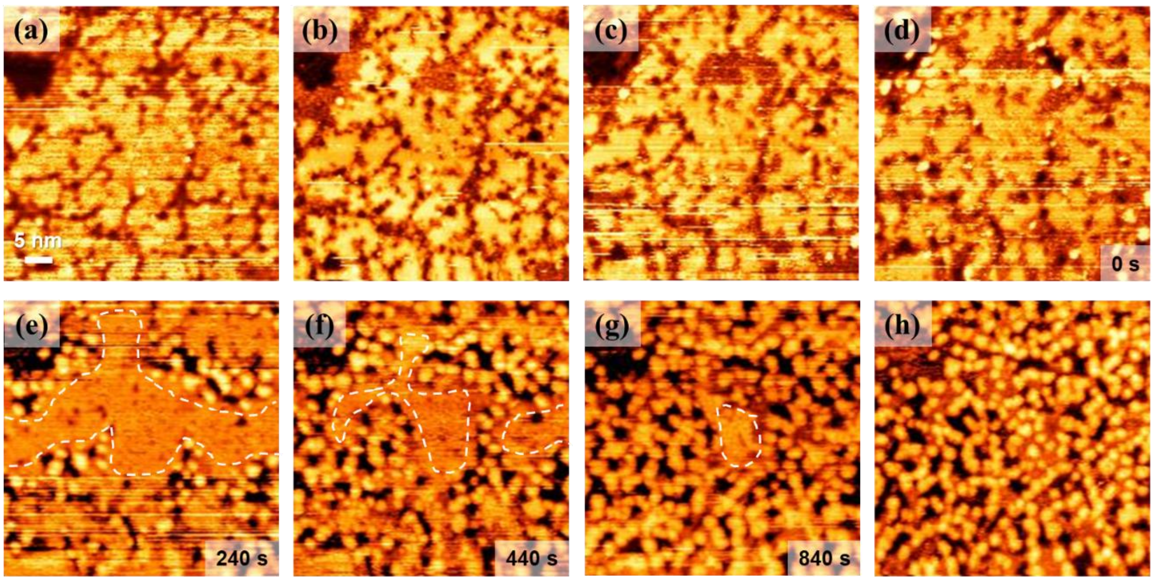

Figure S7. In-situ STM images of water dissociative adsorption on CoO_{2-x}/Pt(111) under near-ambient H_2O atmosphere. STM images of (a) pristine 1 ML $CoO_{1.9}$/Pt(111) in (b) 1×10^{-4}, (c) 2×10^{-2}, (d)-(g) 4×10^{-2} and (h) 2 mbar H_2O at RT (The reaction front in e-g for transformation is marked by white dash line). STM scanning parameters: V_t = 1.25 V, I_t = 0.1 nA.

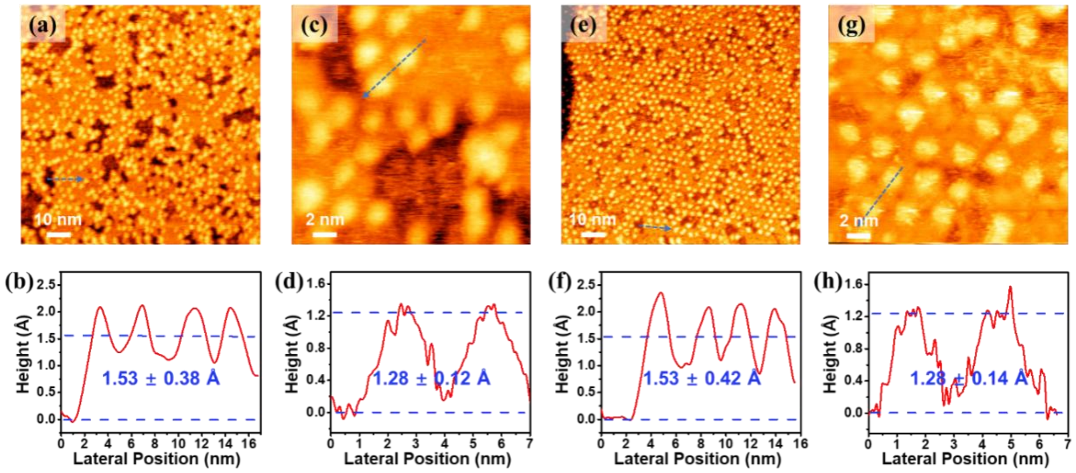

Figure S8. STM images of (a, c) $CoO_{1.9}$/Pt(111) and (e, g) CoO/Pt(111) after exposure to 7 mbar H_2O. STM parameters: V_t = 1.25 V and I_t = 0.1 nA. (b, d, f, h) Line profile along the blue dash arrows in a, c, e, g, respectively.

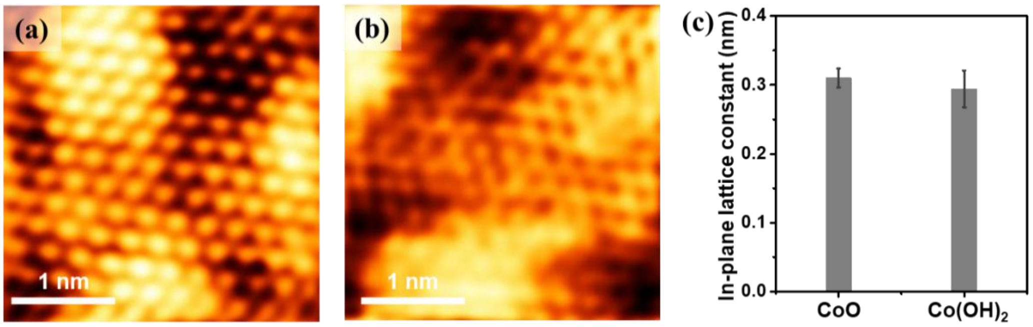

Figure S9. STM images of surface atomic structure of (a) CoO/Pt(111) and (b) $Co(OH)_2$/Pt(111). (c) The surface lattice constants of CoO/Pt(111) and $Co(OH)_2$/Pt(111) films measured in (a) and (b). STM parameter: V_t = 0.2 V, I_t = 0.3 nA.

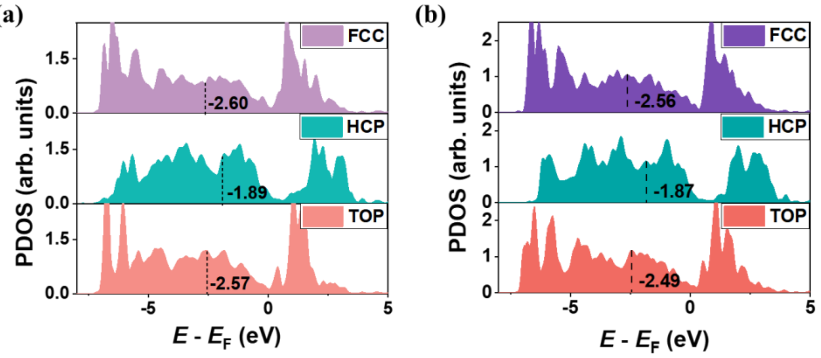

Figure S10. Projected density of states (PDOS) of Co d orbitals calculated using the CoO-(√13 × √13)/Pt(111)-(4 × 4) model (a) and the CoO-(√67 × √67)/Pt(111)-(√84 × √84) model (b). Inserted values indicate the

corresponding d band centers.

Table S1. Calculated results of Bader charges, magnetic moments and oxidation states of Co.

CoO_xH_y	Bader charge (|e|)	magnetic moment (μB)	oxidation state
$Co(OH)_2$	1.31	2.76	2.00
CoOOH	1.55	0.00	3.00
CoO/Pt(111)	1.16	2.57	1.77
$Co(OH)_2$/Pt(111)	1.33	2.72	2.05
CoO_2/Pt(111)	1.39	0.00	2.30

Table S2. Statistical analysis of the XPS fitting results in this work, with binding energies (BE) of specific species and their full-width-at-half-maximum (FWHM) values.

Species		Binding energy (eV)	FWHM
CoO/Pt(111)	surface O	529.6	1.5
	OH	531.2	2
$CoO_{1.9}$/Pt(111)	surface O	529.3	1.5
	interface O	530.2	1.5
	OH	531.7	2

Table S3. The zero-point energy (*ZPE*), integrated heat capacity (δH), entropy correction ($T\Delta S$), and total Gibbs free energy correction (G-E_{elec}) at 298 K for the gaseous molecules. (unit: eV)

Gaseous molecule	*ZPE*	δH	*TS*	G-E_{elec}
O_2	0.10	0.09	0.63	-1.17

Movies

Supplementary Movies S1-S3 provide STM visualizations of the real-time hydroxylation dynamics of ultrathin cobalt oxide films on Pt(111) under H_2O atmospheres across different pressure ranges.

Movie S1. In-situ STM movie showing the real-time hydroxylation dynamics of CoO/Pt(111) under H_2O atmosphere as the pressure increases from 8×10^{-8} to 1×10^{-5} mbar, corresponding to Figure 1.

Movie S2. In-situ STM movie showing the real-time hydroxylation dynamics of CoO/Pt(111) under H_2O atmosphere as the pressure increases from 1×10^{-5} to 2 mbar, corresponding to Figure 2.

Movie S3. In-situ STM movie showing the real-time hydroxylation dynamics of $CoO_{1.9}$/Pt(111) under H_2O atmosphere as the pressure increases from 1×10^{-4} to 2 mbar, corresponding to Figure 4.

References

1. Zeng Z, Chang K-C, Kubal J *et al.* Stabilization of ultrathin (hydroxy)oxide films on transition metal substrates for electrochemical energy conversion. *Nat Energy*. 2017; **2**: 17070.
2. Peterson AA, Abild-Pedersen F, Studt F *et al.* How copper catalyzes the electroreduction of carbon dioxide into hydrocarbon fuels. *Energy Environ Sci*. 2010; **3**: 1311-5.
3. Cramer CJ. Essentials of Computational Chemistry: Theories and Models. *John Wiley & Sons*. 2013.
